# Should our approach to diuretic using in patients with gout change?

**DOI:** 10.1186/s13075-018-1716-7

**Published:** 2018-10-26

**Authors:** Duygu Tecer, Gozde Kubra Yardımcı, Alper Sari, Hakan Babaoglu

**Affiliations:** 1 Rheumatology Department, Mehmet Akif Inan Education and Research Hospital, Ertugrul Street, Sanlıurfa, 63300 Turkey; 2 Department of Internal Medicine, Division of Rheumatology, Hacettepe University, Faculty of Medicine, Ankara, Turkey; 3 Department of Internal Medicine, Division of Rheumatology, Gazi University, Faculty of Medicine, Ankara, Turkey

We read with interest the recent article by Ranieri et al. [1]. The intention of the authors was to investigate the impact of diuretic therapy on the response of urate-lowering drugs (ULDs) in patients with gout. According to the results of this study, diuretic therapy did not impair response to ULDs. Also, there was no significant effect of diuretic use on the achievement of serum urate (SU) targets or on the maximum doses of ULDs. However, it is known that the European League Against Rheumatism (EULAR) has reported its concern in regard to the use of diuretics in patients with gout and recommends substitution of diuretic to losartan or calcium channel blockers if gout is diagnosed in a hypertensive patient receiving loop or thiazide diuretics [2]. For this reason, we believe that the results of this study, which reported the incompatibility with the recommendations of known and accepted organizations, should be interpreted carefully as it may bring a question mark to daily clinical practice.

First, the retrospective design of the study led to the heterogeneity of the patient population. Given the baseline characteristics of enrolled patients, 137 (65.9%) patients in the allopurinol group and 30 (85.7%) in the febuxostat group had hypertension. In addition, 107 (51.4%) patients in the allopurinol group and 20 (57.1%) patients in the febuxostat group had dyslipidemia. But in this study, we did not find any information about the drugs used for hypertension and dyslipidemia which may have an effect on urate metabolism. A large epidemiological study reported that calcium channel blockers and losartan are associated with a lower risk of incident gout among patients with hypertension [3]. It is well known that fenofibrate and statins have uricosuric properties [4, 5].

Second, in the study, alcohol use and dietary habits of patients may directly affect the SU levels. But there is no detailed information on alcohol use and dietary habits of patients. All of these factors may have affected the results of the study.

Lastly, given diuretic indications, 63% of patients using diuretics had pure hypertension without heart failure or chronic kidney disease. In these patients, although there are alternative anti-hypertensive drugs, the reason for the continuation of diuretics after diagnosis of gout is a matter to be discussed.

In conclusion, the results of this study, which can change our clinical practice, consisting of a retrospective and heterogeneous population, should be interpreted with caution. We believe that, as the authors emphasize, there is a need for prospective well-designed studies about the effects of diuretics on the necessary doses of ULDs to achieve the targeted SU level.

## Abbreviations

EULAR: European League Against Rheumatism
SU: Serum urate
ULD: Urate-lowering drug
