# Supplementary material for: Should our approach to diuretic using in patients with gout change?
Source: Arthritis Res Ther. 2018 Oct 26;20:241. doi: 10.1186/s13075-018-1716-7 (PMC6235216; doi:10.1186/s13075-018-1716-7)
Supplement: Related texts

# Reply: Comment on: Should our approach to diuretic use in patients with gout be changed?

**Authors:** Laura Ranieri, Mariano Andres

**Affiliations:** 4 0000 0000 8875 8879 grid.411086.a Rheumatology Department, Hospital General Universitario de Alicante-ISABIAL, Alicante, Spain; 5 0000 0001 0586 4893 grid.26811.3c Universidad Miguel Hernandez de Elche, Alicante, Spain

We thank Tecer and colleagues [6] for their interest and considerations addressed around our article [1]. They discuss whether the approach in the diuretic treatment in patients with gout should be changed according to our results and whether dietary habits or other agents used for hypertension or dyslipidemia might have acted as confounders.

The EULAR recommendations, in both the 2006 and 2016 versions, consider substituting diuretic therapy (loop agents or thiazides) when possible for the management of patients with gout [2, 7]. However, this recommendation was actually based on experts’ opinion (graded as category of evidence 3, grade of recommendation C) and was not unanimous (agreement of 8.2 ± 0.9 out of 10 in 2016). Our findings suggest that this approach may be unnecessary, at least to achieve the proper SU target to ensure crystal dissolution. SU was reduced below 6 mg/dL in the majority of patients (68.1% to 79.1% in the allopurinol group and 78.6% to 81.0% in the febuxostat group) and there were no differences according to diuretics. The rate of patients achieving stricter SU targets was also considerable. Along with this, the dosage of the ULDs was comparable between groups. We agree with the authors that these findings should be replicated in prospective controlled studies, but, in view of our results and recent reports [8], diuretics could be maintained. Certainly, the indication for diuretics may drive the decision. Whether diuretic withdrawal has an impact on future ULD maintenance dosing, in order to prevent the formation of new urate crystals, remains to be explored.

Alcohol and dietary intake were not registered in clinical records, but lifestyle advice is routinely given to all patients with gout and not led by whether they were on diuretics or not. The use of other agents with uricosuric action, such as calcium channel blockers, losartan, atorvastatin, or fenofibrate, was not registered. In our experience, their urate-lowering effect is modest, especially when they are combined with ULDs [9]. Following this, we do not routinely use them when we initiate ULDs. Thus, their impact on our study findings appears to be limited.
